# Process Induced Skin-Core Morphology in Injection Molded Polyamide 66

**DOI:** 10.3390/polym12040894

**Published:** 2020-04-12

**Authors:** Yvonne Spoerer, René Androsch, Dieter Jehnichen, Ines Kuehnert

**Affiliations:** 1 Institute of Polymer Materials, Department of Processing, Leibniz-Institut für Polymerforschung Dresden e. V., Hohe Str. 6, 01069 Dresden, Germany; spoerer@ipfdd.de; 2 Interdisciplinary Center for Transfer-Oriented Research in Natural Sciences, Martin Luther University Halle-Wittenberg, 06099 Halle/Saale, Germany; rene.androsch@iw.uni-halle.de; 3 Institute of Physical Chemistry and Polymer Physics, Department of Nanostructured Materials, Leibniz-Institut für Polymerforschung Dresden e. V., Hohe Str. 6, 01069 Dresden, Germany; djeh@ipfdd.de

**Keywords:** injection molding, crystallization, polarized-light optical microscopy, transmission electron microscopy, X-ray scattering

## Abstract

Polyamide 66 (PA 66) was injection-molded to obtain samples with a structure gradient between skin and core, as it was revealed by analysis of the semi-crystalline morphology using polarized-light optical microscopy (POM). Wide-angle X-ray scattering (WAXS) and small-angle X-ray scattering (SAXS) were employed to characterize thin sections with a thickness in the order of magnitude of 50 µm, allowing detection of crystals of different perfection, as a function of the distance from the surface. It was found that the transparent and non-spherulitic skin layer contains rather imperfect α-crystals while the perfection of α-crystals continuously increases with extending distance from the surface. Since variation of the molding conditions allows tailoring the skin-core morphology, the present study was performed to suggest a reliable route to map the presence of specific semi-crystalline morphologies in such samples.

## 1. Introduction

Aliphatic polyamides (PA) belong to the group of crystallizable polymers with many commercial applications. Their properties depend on the specific semi-crystalline morphology, which develops during melt-processing including, for example, injection molding. Important parameters of the semi-crystalline morphology are the crystal fraction, the crystal structure, habit, size, and orientation. In this study, an analysis of the injection-molding induced morphology of PA 66 was performed, by using polarized-light optical microscopy (POM), transmission electron microscopy (TEM), and X-ray scattering.

During injection molding, crystallization/solidification occurs under influence of shear- and temperature-gradients, which typically leads to the development of different semi-crystalline morphologies between the skin and core [1,2,3]. Molded parts often appear transparent and featureless under an optical microscope in the surface-near regions, which is due to fast cooling of the oriented melt and solidification at rather high melt-supercooling. With increasing distance from the surface, a shear layer/zone is observed, which develops while the molecule segments are subjected to high shear forces during the mold-filling process along the skin layer which has already solidified. Next to the shear layer, towards the center of the cavity, the molecules typically exhibit lower orientation and are cooled more slowly, thus allowing spherulitic crystallization.

For a large number of aliphatic PA, different crystal polymorphs as a function of the conditions of melt-crystallization are reported [4,5,6,7,8,9,10,11,12]. Concerning crystallization of the relaxed melt, a similar crystal/mesophase polymorphism is reported such as for isotactic polypropylene (iPP). Slow cooling results in the formation of α-crystals and spherulites, while fast cooling leads to development of non-lamellar mesophase domains [4,5,6,7,8,9,10,11,12]. However, the critical cooling rate to suppress crystallization at high temperature and crystal perfection is different from iPP. It is worthwhile noting that the distinct high- and low-temperature crystallization processes in PA (6, 66, and 11) have been associated with different mechanisms of crystal nucleation [13,14], ultimately yielding to qualitatively different semi-crystalline morphologies, and having impact on, for example, mechanical and optical properties [15].

Prediction of structure formation of injection molded parts requires knowledge of the time- and position dependent temperature and shear-rate profiles and knowledge of the solidification/crystallization process at such conditions. Recent advances in this field have been achieved by application of fast scanning chip calorimetry (FSC). This method allows the mimicking of crystallization at cooling conditions that are present in injection molding, that is, at cooling rates of the order of magnitude of hundreds of K/s [16]. In conjunction with X-ray analyses information about the formation of specific crystal polymorphs can even be obtained [9,11,17]. Recently, the cooling-rate profile during low-shear injection molding of poly(butylene terephthalate) (PBT) was simulated in the skin and core regions and correlated with experimental quiescent-crystallization data obtained by FSC, yielding a very good prediction of the crystallinity difference in the various regions of the molded part [18]. In the present work, we attempt to expand our efforts in characterization and prediction of structure formation in injection molding of polymorphic materials by using the example of PA 66.

It is the aim of this study to provide an experimental route to identify the process-induced morphology in terms of crystal shape and structure, orientation, crystallinity, and spherulitic superstructure in injection molded parts as a function of the distance from the surface and to correlate them with the microstructure assessed by optical microscopy. To achieve this aim a method was developed to investigate the process induced morphology layer by layer. With this research we follow prior and similar work performed for example on PA 6 and 66 [19,20,21] but also iPP [1,22]. However, we attempt to go beyond existing knowledge by interpretation of results in the context of our recent studies about crystallization at processing-relevant cooling conditions.

## 2. Materials and Methods

### 2.1. Material

A commercial injection-molding grade of PA 66 was used, as received from BASF (Ludwigshafen, Germany). The material had a melt flow index of 115 cm³/10 min (275 °C/5 kg, ISO 1133). It was non-nucleated, unfilled, and natural colored.

### 2.2. Processing

**Figure 1:**
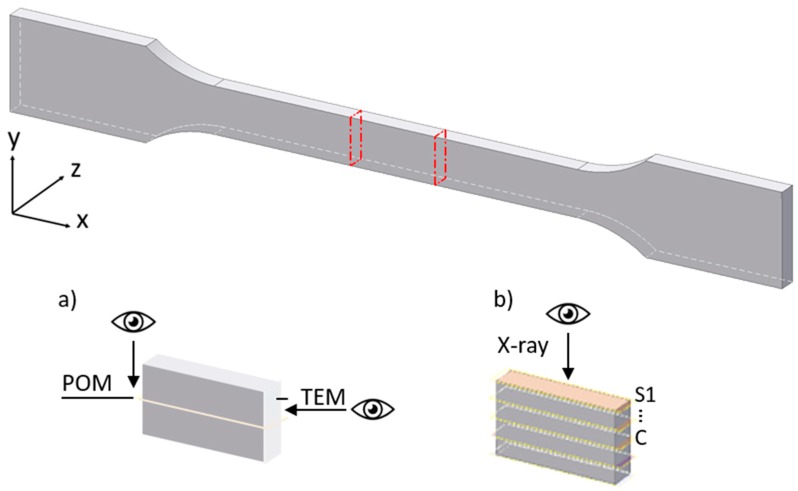
Tensile test bar type 1A. Preparation scheme: (**a**) for polarized-light optical microscopy (POM) and transmission electron microscopy (TEM), (**b**) for X-ray scattering.

Before processing, the material was dried under vacuum at 80 °C for 12 h. Tensile test bars (DIN EN ISO 527, Type 1A) with a cross-section of 10 mm × 4 mm, shown in Figure 1, were injection molded. The barrel and mold temperatures were 300 and 40 °C, respectively, the injection speed was 40 mm/s, the holding pressure was 70 bar and the cycle time was 58 s.

### 2.3. Sample Preparation

After processing, the samples were prepared for morphological studies. As shown in Figure 1, a sample piece was removed from the tensile bar center. From this sample, thin sections corresponding to Figure 1a,b were removed using a rotary microtome at −30 °C, equipped with a tungsten-carbide knife. The thin sections were taken from different parts of the sample depending on the characterization method used. The marked eyes in Figure 1 indicate the viewing direction from which the sample was analyzed with respect to the used methods. For POM investigations, 10 µm thick thin sections in flow direction were taken from the sample center (Figure 1a). For TEM, the cross-section of the sample was trimmed (Figure 1a) and contrasted with a formaldehyde/osmium tetroxide solution. For X-ray scattering, 50 µm thick thin sections were taken layer by layer from the skin (S) to the core (C) parallel to the flow direction (Figure 1b).

### 2.4. Polarized-Light Optical Microscopy (POM) and Transmission Electron Microscopy (TEM)

The POM images were taken by an Axio Imager from ZEISS (Oberkochen, Germany) with an objective of 50× magnification. Images of the skin and core areas of the cross-section were collected by using a TEM Tecnai G2 from FEI-Company (Hillsboro, OR, USA), which now belongs to Thermo Fisher Scientific (Waltham, MA, USA). An acceleration voltage of 200 kV was used.

### 2.5. X-ray Scattering

X-ray analyses in different scattering ranges were performed on thin sections, which were cut layer by layer from the skin to the core, parallel to the direction of flow (see Figure 1b). Wide- and small-angle X-ray scattering (WAXS and SAXS, respectively) experiments were conducted consecutively by means of the multirange device Ganesha 300 XL+ (SAXSLAB ApS, Copenhagen, Denmark) equipped with a µ-focus X-ray tube and a Göbel mirror, providing monochromatic, parallel-beam Cu Kα radiation. Scattering intensities were measured by a two-dimensional (2D) Pilatus 300K detector (DECTRIS Ltd., Baden-Daettwil, Switzerland). The experiments were done in vacuum, in asymmetric-transmission mode, that is, the sample surface was oriented perpendicular to the primary beam. To connect WAXS and SAXS, the raw data were corrected for absorption (primary intensities in different measuring configurations were adjusted by using the sample transmission values). The results are presented as 2D scattering patterns and 1D line scans (radial scattering curves).

For calculation of the crystallinity, X_C,_ based on the intensity parts scattered by crystalline as well as amorphous amounts, a peak-fitting with pseudo-Voigt functions were executed by using the software package Analyze (part of the RayfleX software package for X-ray scattering devices; GE Sensing and Inspection Technologies GmbH, Ahrensburg, Germany). The minor anisotropies due to processing were neglected in the calculation of crystallinity. The obtained full widths at half maximum (FWHM) of selected scattering peaks served as basis to calculate average crystallite sizes 〈L〉_hkl_ perpendicular to the related lattice plane (hkl) by using the Scherrer equation [23]. Long periods, D_L,_ were derived from the maximum position in the SAXS curve I(q) by using the Bragg equation [24] (after background correction).

## 3. Results and Discussion

**Figure 2:**
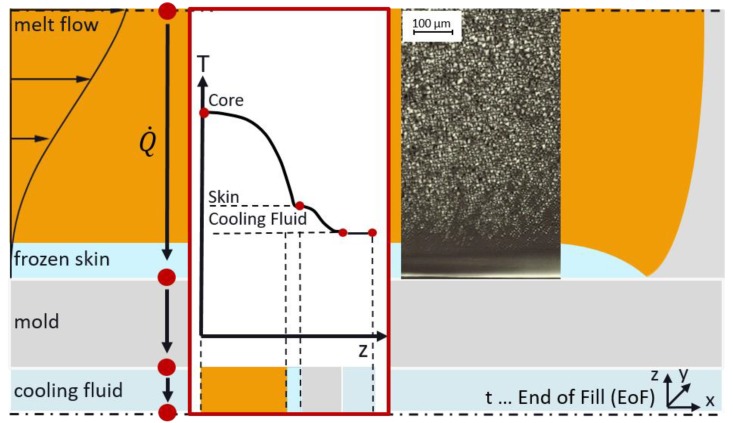
Melting flow scheme during injection-molding. The melt flow inside the cavity with the temperature curve from the core to the skin at the end of filling stage.

During injection molding, the process parameters, especially the temperature profile, have a significant influence on the morphology of the moldings. It is known from the literature [25,26], that the melt forms a fountain flow as it flows into the cavity. Within this fountain flow, the melt moves from the inner core out to the cold mold wall. When the melt gets into contact with the mold wall, the melt solidifies due to the high temperature difference between the melt and the mold, as shown in Figure 2.

**Figure 3:**
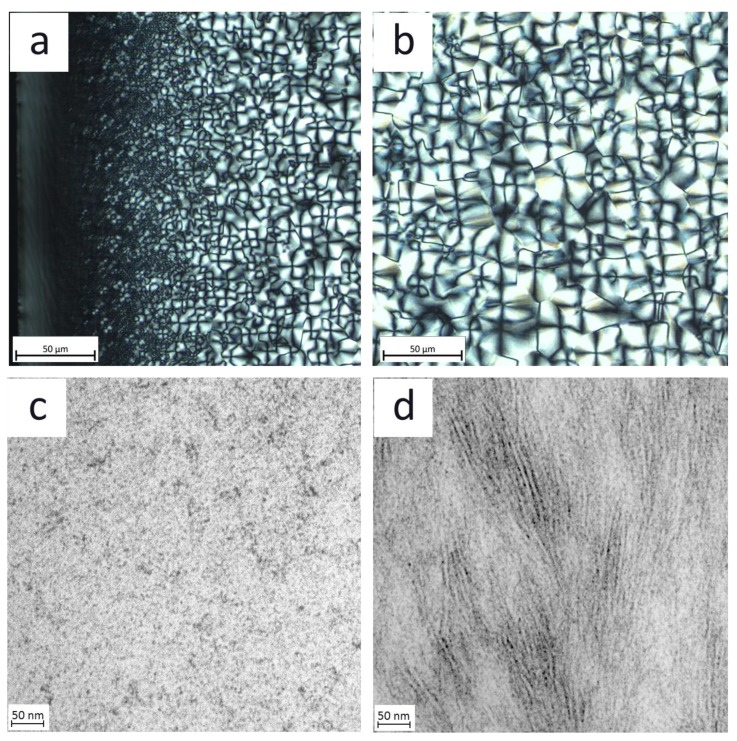
Optical analysis: POM micrograph of: (**a**) the skin region with increasing spherulite size and (**b**) of the core region. TEM micrograph of: (**c**) the skin region with contrast-related artefacts and (**d**) of the core region with oriented lamellae with a thickness of 5.1 nm.

The optical analysis of the PA 66 sample revealed the significant/notable influence of the temperature difference between the melt and the mold (220 K) on the morphology gradient from the skin to the core inside the molded part. As can be seen by the POM images of Figure 3a,b, a skin layer of about 50 µm thickness was observed, mainly due to the high temperature difference between the melt and the mold of 260 °C. Inside the skin layer, structural heterogeneities are not visible. At 50 µm distances from the mold, in the direction towards the core, spherulites are visible, increasing in their size with increasing distance from the skin. The visualization of crystalline structures is limited by the low resolution of POM, and therefore TEM was employed to gain further information about the crystal morphology. Figure 3c,d show TEM images obtained from the skin and core. At the skin, no heterogeneities are observed whereas in the core, lamellae with a thickness of 5.1 nm are visible (average of 10 lamellae). Therefore, Figure 3 illustrates the strong influence of the thermal process history on the formation of the semi-crystalline structures during the injection-molding process. No visible crystalline structures could be detected in the skin layer. In contrast, behind the skin layer towards the core, spherulites could be observed increasing in size. The morphology in the core depends on the heat transfer through the solidified skin layer to the mold. Parameters with the greatest influence, are the thermal conductivity of the material (0.23 W/(K·m) [27]), the temperature difference between the melt and the mold and the flow velocity of the melt, and therefore the resulting thickness of the skin layer. By considering the high temperature difference of 220 K between melt and mold and taking into account the literature [12,13,14,15,16,17,18,19,20], it is assumed that cooling in the core is in the order of 400–600 K/s slower than at the skin. Accordingly, inside the core, the chains have sufficient time to form crystals and arrange in a spherulitic superstructure.

**Figure 4:**
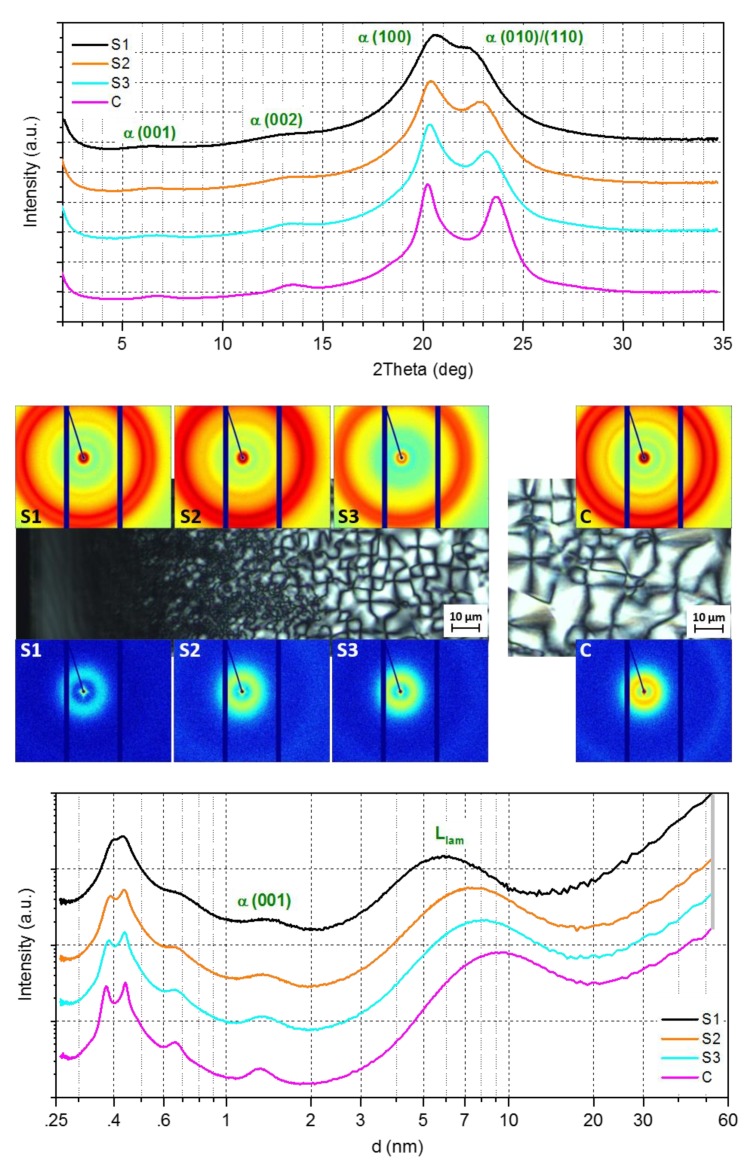
X-ray analysis from skin to core. 2D-Wide-angle X-ray scattering (WAXS) above and 2D-small-angle X-ray scattering (SAXS) patterns below the POM micrographs in the middle. WAXS and SAXS patterns were taken at the sample positions as visualized inside the POM micrographs. Radial intensity graphs 1D-WAXS (above: I vs. 2θ) and 1D-WAXS/SAXS (below: lg I vs. lg d), azimuthally integrated from 2D-WAXS and 2D-SAXS patterns (curves are shifted vertically). Scattering area coordinates: 2D-WAXS – q_x_: −16.8–20.0 nm^−1^, q_y_: −17.5–13.0 nm^−1^, and 2D-SAXS – q_x_: −4.19–5.34 nm^−1^, and q_y_: −4.31–3.14 nm^−1^.

**Figure 5:**
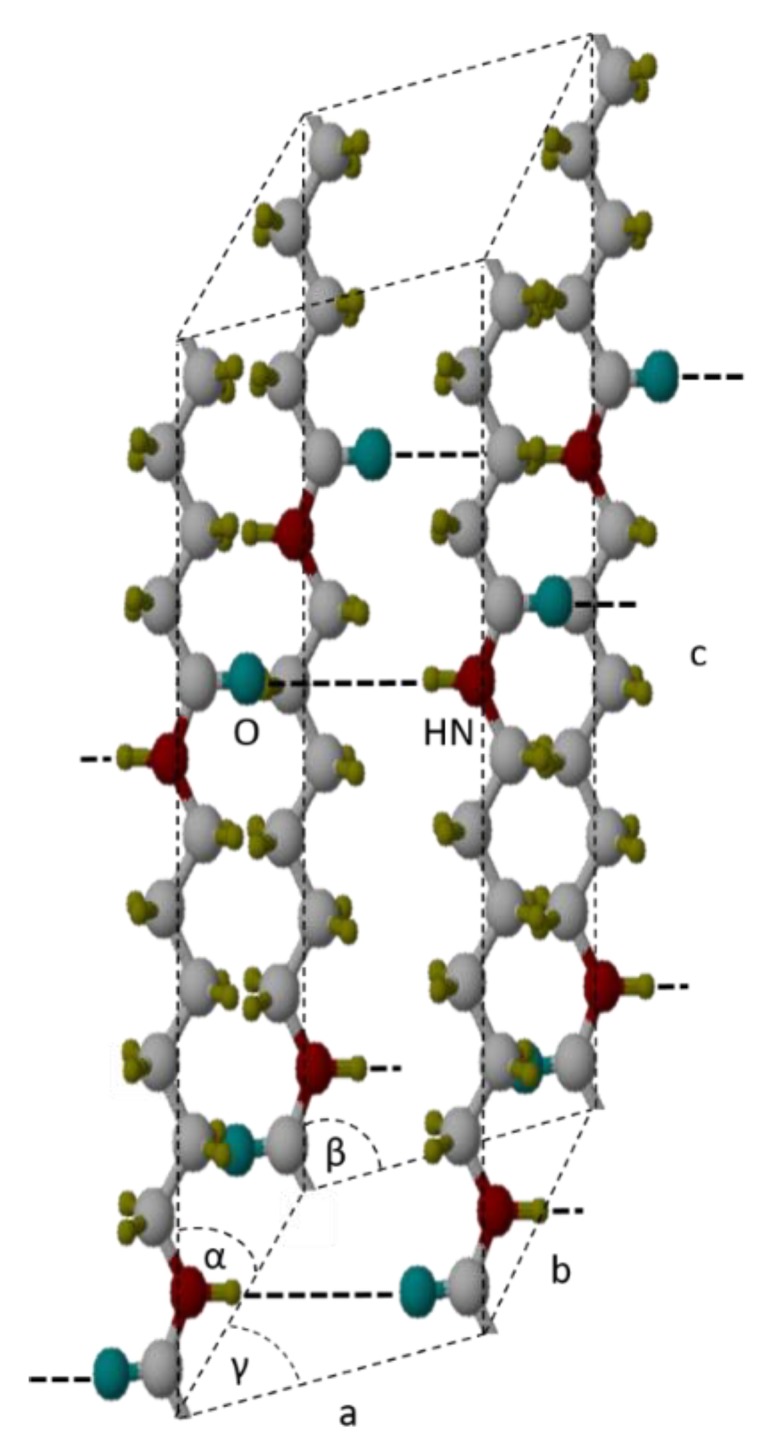
Chain orientation along a-, b-, and c-axes within a triclinic crystalline unit cell of polyamide 66 (PA 66). (white … carbonate, blue … oxygen, red … nitrogen, yellow … hydrogen).

The results of X-ray scattering experiments are shown in Figure 4. Samples with a thickness of 50 μm were cut from the injection molded test specimen such that they are perpendicular to the samples which were analyzed using POM as is shown in Figure 1. The WAXS and SAXS patterns are shown such that their position corresponds to the appropriate position on the POM micrograph. The azimuthally integrated radial scattering curves are shown above and below, respectively. The WAXS curves feature peaks at spacing of 1.4, 0.66, 0.43, and 0.38 nm caused by scattering at the (001), (002), (100), and (010)/(110) lattice planes, indicating the presence of the triclinic α-modification with the unit cell (shown in Figure 5) parameters a = 0.49 nm, b = 0.54 nm, c = 1.72 nm (chain direction), α = 48.5°, β = 77°, and γ = 63.5° [28]. The 2D-pattern revealed that the crystals are weakly oriented (negligible in the sense of discussion). Most important, however, is the observation that the maxima of the (100) and (010/110) peaks change their position as a function of the distance from the surface.

According to the literature [28], the angular distance between the (100) and (010)/(110) diffraction maxima is proportional to the crystal perfection. A so-called crystal perfection index (CPI) can be calculated according to Equation (1) [29]:

(1) $$CPI\;\left[\%\right]=\frac{\left(d_{100}/d_{010}\right)-1}{0.189}\times 100$$

where d_100_ is the interplanar spacing of α-(100) planes and d_010_ of α-(010)/(110) planes. The denominator correlates to the distance in a well-crystallized sample (with CPI = 100%), reported in [27,28]. Table 1 shows the CPI values at different distances from the skin. As such, the crystal perfection increases from skin to core up to 90%. The WAXS data were also used to calculate the crystallinity of the core and the skin-layer. The former is about 37%, whereas the latter is, in contrast, much lower at 25%.

In PA 66, a lamellar morphology of alternating crystalline and amorphous sublayers can be assumed, which cause an interference maximum (long period D_L_) in the SAXS region. Long periods of 9.2 and 5.8 nm were estimated for the core and skin, respectively. Thus, a linear crystallinity model (two-phase model) can be applied. Additionally, supposing that the interface between the sublayers and the (001) net plane are nearly parallel to each other (due to the hydrogen bridges), the crystallite sizes (Scherrer) 〈L〉_001_ and 〈L〉_002_, respectively, can be compared with the thickness l_C_ of the crystalline sublayer. Consequently, the crystallized polymer chains continue tilted through its sublayers. Finally, the relation l_C_ = D_L_ × X_C_ (with X_C_ as crystallinity) can serve as unbiased estimate. The trend of the l_C_ values is given in Table 1. Inside the error limits, the calculated l_C_ values follow the trend as shown of D_L_.

## 4. Conclusions

For understanding and predicting of the structure formation of melt-processed polymer materials, a detailed knowledge of phase transitions is necessary in well-resolved scales of position, time, and temperature. In this paper, the semi-crystalline morphology of injection molded PA 66 was investigated in terms of the influence of cooling conditions during the manufacturing of test specimens. Here, the results, achieved ex-situ after completion of manufacturing, were presented. The investigations have shown that the thermal history, especially during the filling phase, has a strong influence on the morphology in the skin and core region. With decreasing of the cooling rate from skin to core, the spherulite sizes increase. This was accompanied by an improved perfection of the crystalline phase inside the core, where a triclinic α-phase structure existed/formed. In comparison to the core, at the skin, the rapid cooling when the melt got into contact with the cold mold surface hindered the formation of spherulites. However, the results of X-ray analysis show that the skin layer contains a rather poor crystalline α-phase, which confirms that crystalline fractions can also form under process conditions which promote rapid cooling of the material. This knowledge can help to direct the processing of injection-molded construction materials to improve their application properties. A detailed study of structure–property relationships can be the basis for fabricating tailor-made materials.

## Figures and Tables

**Table 1:** Determined values for selected reflections: position, perfection, crystallite size, crystallinity, long period, and crystalline sublayer (WAXS and SAXS results).

Sample Position	Reflections		Crystal Perfection Index	Mean Crystallite Sizes (Scherrer)		Crystallinity	Long Period	Crystalline Sublayer (l_C_ = D_L_ × X_C_)
	d (nm)		CPI (%)	〈L〉_hkl_ (nm)		X_C_ (%)	D_L_ (nm)	l_C_ (nm)
	d_100_	d_010/110_		〈L〉_002_	〈L〉_100_			
S1	0.4335	0.3942	52.7	2.2	4.7	25.2	5.8	1.5
S2	0.4367	0.3852	70.7	2.9	6.4	36.6	7.3	2.7
S3	0.4377	0.3813	78.3	2.9	7.4	37.8	7.9	3.0
C	0.4397	0.3754	90.6	4.0	9.3	37.3	9.2	3.5
Errors ± Δx	~0.0005	~0.0010	~1.6	~1.2	~0.8	~1.5	~0.1	~1.3
